# Genetic mapping of a male factor subfertility locus on mouse chromosome 4

**DOI:** 10.1007/s00335-018-9773-4

**Published:** 2018-08-31

**Authors:** Hideo Gotoh, Ikuo Miura, Shigeharu Wakana

**Affiliations:** 1 Division of Animal Sciences, Reproductive Biology Unit, Institute of Agrobiological Sciences, NARO, 1-2 Owashi, Tsukuba, Ibaraki 305-8634 Japan; 2 Technology and Development Team for Mouse Phenotype Analysis, RIKEN BioResource Research Center, 3-1-1 Koyadai, Tsukuba City, Ibaraki 305-0074 Japan; 3 Department of Gerontology, Institute of Biomedical Research and Innovation, 2-2 Minatoshima Minamimachi, Chuo-ku, Kobe-city, 650-0047 Japan

## Abstract

Male reproductive anomalies are widely distributed among mammals, and male factors are estimated to contribute to approximately 50% of cases of human infertility. The B10.M/Sgn (B10.M) mouse strain exhibits two adverse reproductive phenotypes: severe teratospermia and male subfertility. Although teratospermia is known to be heritable, the relationship between teratospermia and male subfertility has not been well characterized. The fertility of B10.M male mice is considerably lower (~ 30%) than that of standard laboratory mouse strains (~ 70%). To genetically analyze male subfertility, F2 males were produced by intercrossing the F1 progeny of female B10.M and male C3H/HeN mice. The fertility of each F2 male mouse was assessed based on the outcomes of matings with five females. Statistical analysis of correlations between the two reproductive phenotypes (teratospermia and subfertility) in F2 males (*n* = 177) revealed that teratospermia is not the cause of male subfertility. Quantitative trait loci (QTL) analysis of the male subfertility phenotype (*n* = 128) using GigaMUGA markers mapped one significant QTL peak to chromosome 4 at 62.9 centimorgans (cM) with a logarithm of odds score of 11.81 (P < 0.05). We named the QTL locus *Mfsf1* (male factor subfertility 1). Further genetic analysis using recombinant males restricted the physical area to 1.53 megabasepairs (Mbp), encompassing 22 protein-coding genes. In addition, we found one significant QTL and one indicative QTL on chromosome 5 and 12, respectively, that interacted with the *Mfsf1* locus. Our results demonstrate that genetic dissection of male subfertility in the B10.M strain is a useful model for characterizing the complex genetic mechanisms underlying reproduction and infertility.

**Electronic supplementary material:**

The online version of this article (10.1007/s00335-018-9773-4) contains supplementary material, which is available to authorized users.

## Introduction

Human infertility is a common problem worldwide, and male factors are estimated to contribute to 50% of cases (Agarwal et al. 2015; Melodie and Christine 2018). The etiology of male infertility is highly variable and has been associated with endocrinological, immunological, neurogenic, and environmental factors (Iammarrone et al. 2003). A correlation between male infertility and emergence of cancer has also been reported (Nagirnaja et al. 2018). Although genetic contribution to male factor infertility are evident in humans, limited associations with chromosomal alterations, such as Yq microdeletions and Klinefelter’s syndrome, and mutations in specific genes, such as CFTR, have been reported (Neto et al. 2016). Studies in mice have identified 666 genes that cause male infertility when disrupted (Nagirnaja et al. 2018). As genomic information continues to accumulate, an increasing number of genetic variations linked to male infertility are expected to be identified, and the search for novel genetic variations that underlie male factor infertility is already underway (Miyamoto et al. 2017; Robay et al. 2018; Halder et al. 2017).

Subfertility is defined as reproductive efficiency that lies between that of infertility and normal reproductive performance. This low efficiency is a major concern in agriculture and animal production. For example, in dairy cows, a trend of declining fertility persists for several decades (LeBlanc 2010). Substantial attention has been paid to understand the correlation between milk production and cow fertility (Jamuna and Chakravarty 2016; Berry et al. 2016). With the advent of newly developed technologies in genome science, application of genomic information to improve fertility has become a promising mechanism to improve unfavorable reproductive conditions (Taylor et al. 2018).

Use of model mouse strains to characterize fertility is advantageous because the effects of genetic background can be assessed in a reproducible manner. For example, the well-characterized B10.M/Sgn mouse strain is characterized by male subfertility and severe teratospermia, with ~ 60% of sperm exhibiting abnormal morphology (Gotoh 2010). Previously, we reported that the teratospermia phenotype is heritable (Gotoh et al. 2012) and mapped two of the loci responsible: *Shm1* on chromosome 1 and *Shm2* on chromosome 4. Interactions between these two loci were evident. Homozygosity of the *Shm1*^*B10.M*^ allele was required to express teratospermia, and the enhancing effects of the *Shm2*^*B10.M*/*B10.M*^ genotype were observed only when the genotype of the animal was homozygous for the *Shm1*^*B10.M*^ allele. However, whether a link exists between the teratospermia phenotype and male subfertility in this mouse strain has not previously been investigated. Therefore, in the present study, we performed genetic analyses on the male subfertility phenotype and explored linkage with the teratospermia phenotype.

## Materials and methods

### Animals

All experiments were approved by the Institutional Animal Care and Use Committee of the Institute of Agrobiological Sciences. Animals were housed and cared for according to guidelines established by the Committee. C3H/HeNCrlCrlj (C3H) mice were purchased from Charles River Japan (Yokohama, Japan). C57BL/10J (B10) mice were purchased from S. L. C. (Hamamatsu, Japan). B10.M/Sgn (B10.M) mice are maintained at our facility. Animals were maintained on a cycle of 12 h of light and 12 h of darkness under specific-pathogen-free conditions. The commercial mouse diet CRF-1 (Charles River Japan, Yokohama, Japan) and water were provided.　F2 animals were produced by intercrossing F1 animals obtained from crossing B10.M females with C3H males. Recombinant F2 males in which recombination occurred between the *D4Mit251, D4Mit54*, and *D4Mit170* microsatellite markers were selected for fine mapping of the locus responsible for the male subfertility phenotype detected by QTL analysis.

### Male fertility analysis

Mature, virgin, C3H females ages 8–12 weeks were used for the assay. Five female mice were mated with each male mouse. Male fertility is assessed according to a standard methodology for reproductive toxicological assay (Teramoto et al. 1980; Mitchard et al. 2012). Two weeks after the vaginal plug was observed, the female mice were dissected. The numbers of corpus luteum (yellow body) on ovaries and the numbers of embryos in uterus were counted under a stereo microscope (SMZ25; Nikon, Tokyo). Because yellow body is the remains of ovarian follicle that has released a mature ovum during a previous ovulation, the ratio of the total number of embryos to the number of yellow bodies was used to estimate the fertility percentage for each mating. The fertility of each male mouse was expressed as the mean fertility percentage for the five matings.

### Sperm morphology test

Sperm samples were collected from 3- to 5-month-old male mice after fertility testing was completed. Sperm morphology was analyzed as described earlier (Gotoh 2010). Two independent samples, each containing a minimum of 200 sperm cells, were analyzed under 400 × magnification using a differential interference contrast microscope (DMRXA2; Leica Microsystems; Cambridge, UK).

### Quantitative trait loci (QTL) analysis

Genomic DNA samples were prepared from mouse tail snips (~ 5 mm) as previously described (Hirawatari et al. 2015b). Samples from 128 F2 animals were genotyped using GigaMUGA markers (Morgan et al. 2015) at the Genetics Laboratory, University of North Carolina, Chapel Hill. QTL analysis was performed using R/qtl software (Broman et al. 2003; Broman and Sen 2009). The *scanone* function was used for single QTL analysis, and the *scaonetwo* function was used to detect epistatic interactions between two loci. The threshold for each assay was determined by permutation tests (*n* = 1000). *P* values less than 0.05 and 0.37 were considered statistically significant and suggestive, respectively, for QTL analyses.

### Database search

Information of position, MGI ID, feature type, and symbol of genes is referred to the Mouse Genome Informatics (MGI) website (http://www.informatics.jax.org/marker). Information of known reproductive phenotype of genes is referred to the International Mouse Phenotyping Consortium (IMPC) website (http://www.mouse.phenotype.org). Information of expression of genes in testis is referred to the Expression Atlas database of the European Bioinformatics Institute website (http://www.ebi.ac.uk/gxa.home). Information of single nucleotide polymorphism (SNP) of genes among C3H/He, C3H/HeJ, C57BL/10J, C57BL/6, and C57BL6J inbred strains is referred to the SNP database of the MGI website (http://www.informatics.jax.org/snp). All information was updated on July 31, 2018.

### Statistical analyses

Correlations between male subfertility and teratospermia phenotypes were analyzed using Spearman’s rank correlation test. Comparisons between groups were analyzed by one-way analysis of the variance (ANOVA) using SPSS 16.0 for Windows (Analytical Software; Chicago, IL, USA).

## Results

### Male subfertility and teratospermia

**Fig. 1:**
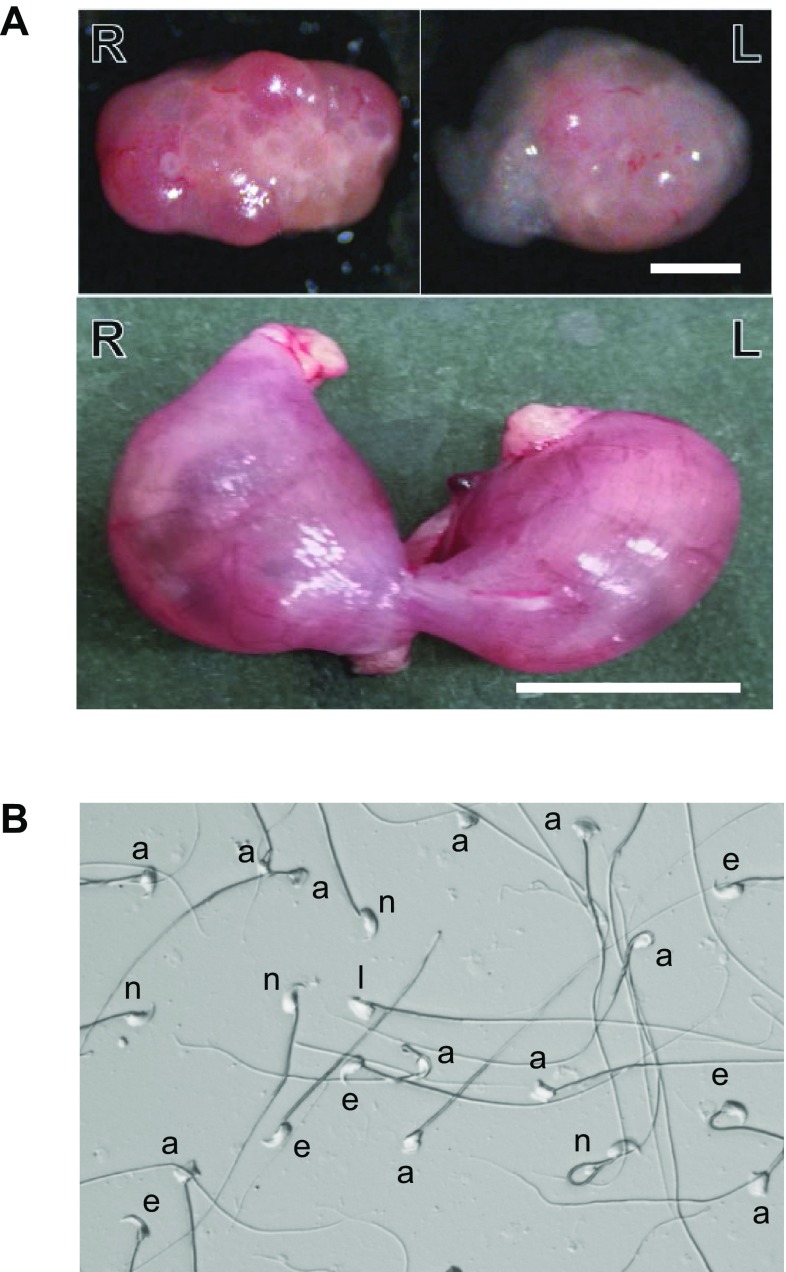
Abnormal male reproductive phenotypes present in the B10.M strain. **a** A representative example of the fertility assay used in this study showing subfertility of an F2 male. Upper photos show a pair of ovaries. The right (R) ovary contains eight yellow bodies, and the left (L) ovary contains one yellow body. Horizontal bar, 100 μm. Lower photo shows a uterus containing fetuses on 13th day of pregnancy. Each side of the uterus contains one fetus, respectively. The fertility was calculated to be 22% in this case. Horizontal bar, 10 mm. **b** An example of teratospermia of an F2 male. Abnormal sperm head morphology is shown. *n* represents a normal spermatozoon; *a* shows ectopic attachment of the flagellum; *a* shows a spermatozoon with amorphous head, and *l* shows the lack of the usual hook

Figure 1a shows a representative example of the ovaries and a uterus from a C3H female mated with a sub-fertile F2 male. In this case, nine yellow bodies and two embryos were present, resulting in a fertility estimate of 22%. Figure 1b presents an example of a sperm spread from an F2 male exhibiting severe teratospermia (64% abnormal spermatozoa) with a number of morphological abnormalities.

### Male subfertility is not correlated with teratospermia in mice

**Fig. 2:**
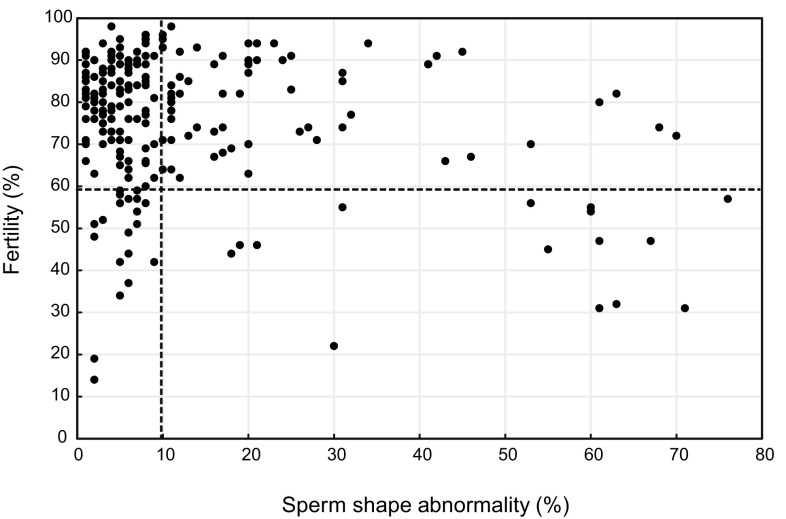
Dot matrix evaluating the relationship between teratospermia and male subfertility phenotypes. Each dot represents the sperm shape abnormality and fertility scores for one F2 male. The horizontal-dotted line indicates a rough threshold to differentiate between normal and low fertility (58.7%; mean − SD). The vertical-dotted line indicates a rough threshold to differentiate between normal and high frequencies of abnormal sperm morphology (9.9%; mean + SD). A statistically significant correlation was not identified by Spearman’s correlation test (*P* > 0.05)

The correlation between male fertility and sperm morphological abnormalities in F2 males is shown in Fig. 2. The horizontal dotted line at 58.7% represents the statistical mean fertility minus the standard deviation (SD) for B10 males (*n* = 10). The vertical-dotted line at 9.9% represents the statistical mean percentage of sperm shape abnormalities plus the SD for B10 males (*n* = 10). These lines define approximate boundaries between high and low values for each phenotype. A considerable number of individuals are found in both the upper-right square and the lower-left quadrants. Males plotted in the upper-right quadrant exhibit normal fertility with teratospermia. Males plotted in the lower-left quadrant exhibit subfertility with normal sperm morphology. Statistical analysis of F2 males (*n* = 177) confirmed the lack of a correlation between the two phenotypes (*P* > 0.05).

### QTL mapping of a male factor subfertility locus

**Fig. 3:**
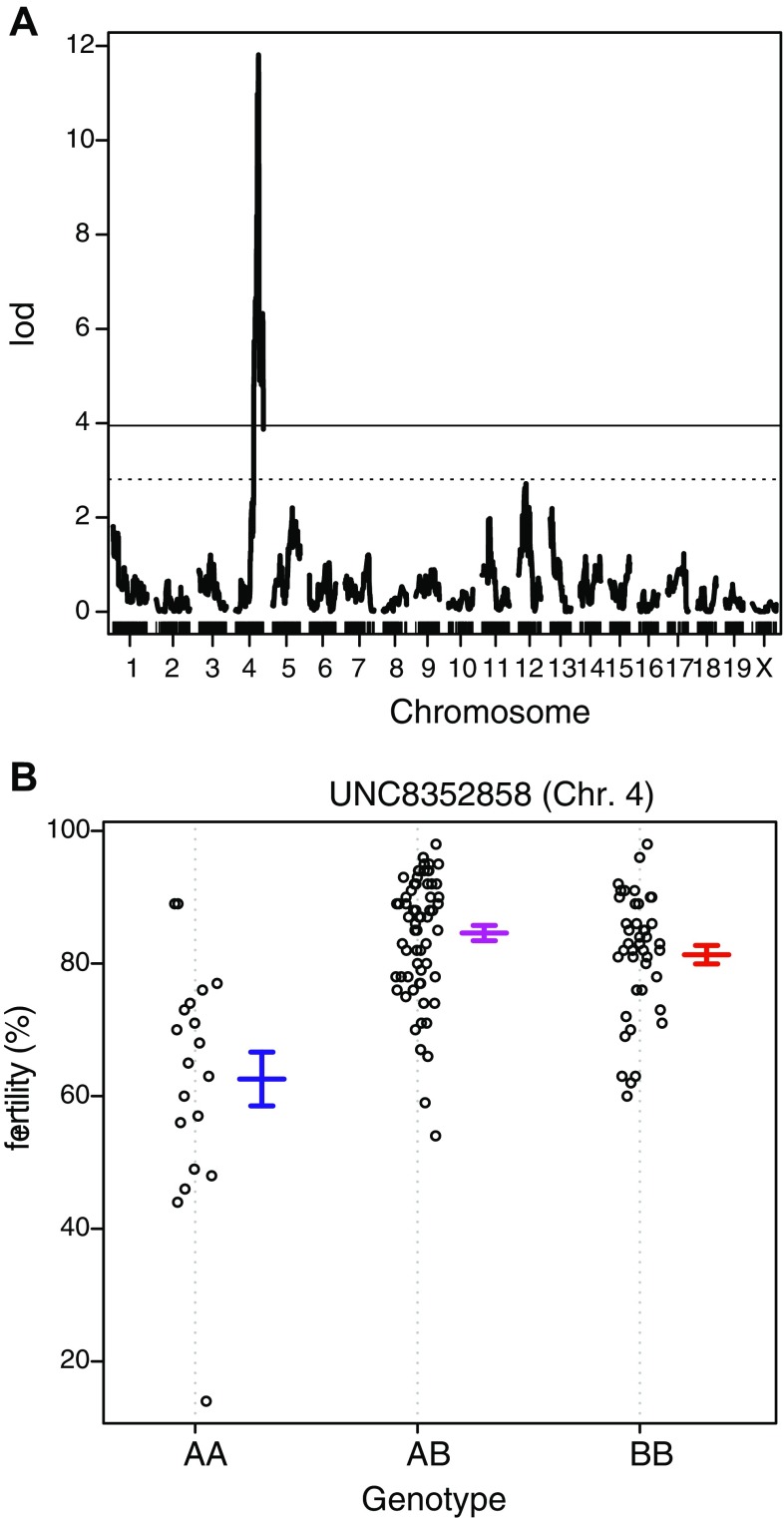
QTL analysis of the male subfertility phenotype in the B10.M strain. **a** One significant peak (LOD score, 11.81) is located on chromosome 4 at 62.94 cM. The horizontal solid line (LOD score, 3.97) and dotted line (LOD score, 2.83) indicate thresholds for statistical significance (*P* > 0.05) and suggestiveness (*P* > 0.37), respectively. The LOD score for the QTL peak identified on chromosome 12 is 2.72. The thresholds were determined by permutation testing (*n* = 1000). **b** Effect of *UNC8252838* genotype on male fertility. The marker nearest the QTL peak on chromosome 4 is *UNC8352838*. Each circle indicates the fertility of a male. The mean fertility ± standard error for each genotype is shown to the right of the circles. *AA* indicates homozygosity for the B10.M allele, *AB* indicates heterozygosity, and *BB* indicates homozygosity for the C3H allele

The *scanone* function of the R/qtl package revealed one significant QTL peak on chromosome 4 at 62.94 cM with an LOD score of 11.81 (Fig. 3a). We tentatively named this locus male factor subfertility 1 (*Mfsf1*). These results confirmed that male subfertility is heritable in the B10.M strain. The marker nearest the QTL peak is *UNC8352858*. The effects of the *UNC8352858* genotype on male fertility are shown in Fig. 3b. Chromosome interval mapping using the R/qtl package estimated that the *Mfsf1* locus mapped between the *UNCHS013009* (59.84 cM) and *UNCHS013136* (63.47 cM) markers (*P* < 0.05).

### Genetic mapping of the *Mfsf1* locus

**Fig. 4:**
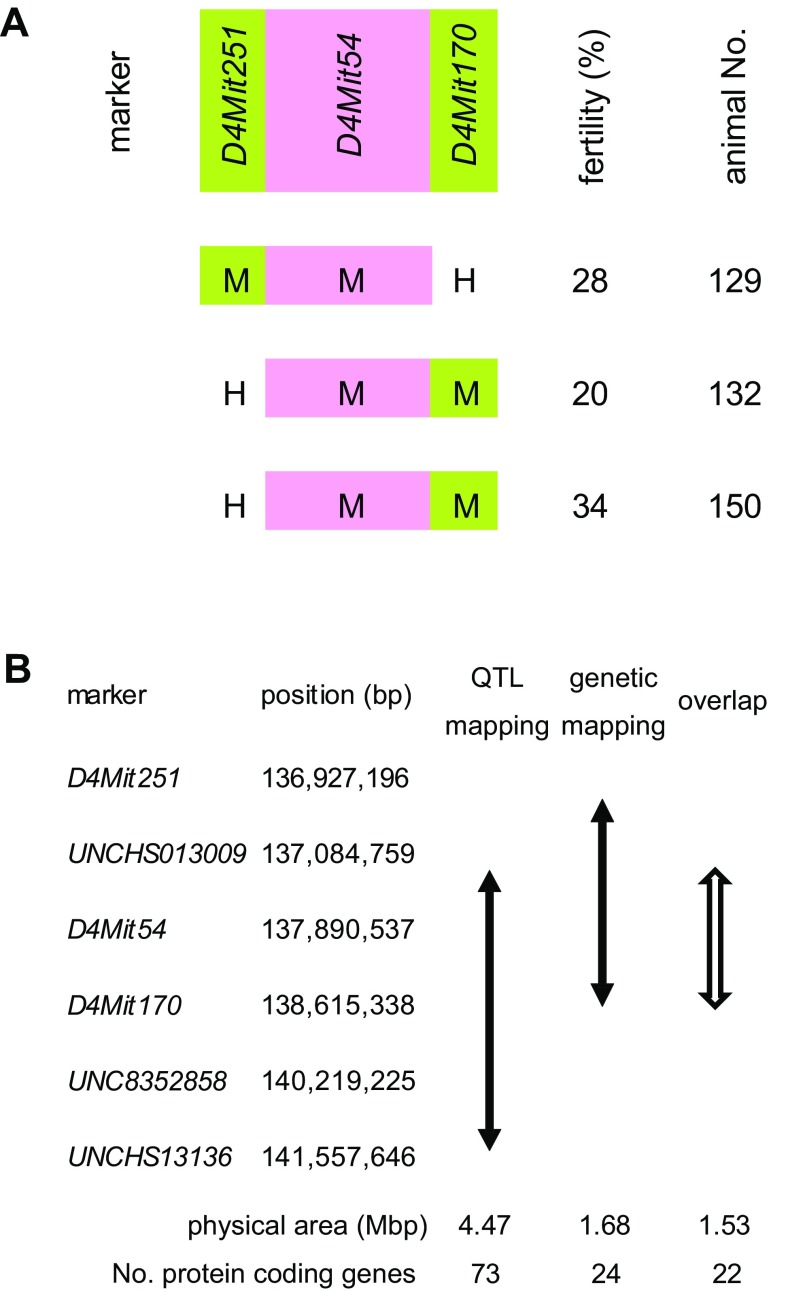
Genetic mapping of the *Mfsf1* locus within the QTL interval on chromosome 4 using recombinant males. **a** Genotypes *M* and *H* indicate homozygosity and heterozygosity of the B10.M allele, respectively. The *pink* and the *light green* regions originated from B10.M chromosomes indicate the presence and absence of the *Mfsf1* locus, respectively. The observed fertility and the identification number of the animal are listed to the right. **b** Comparison of mapping of the *Mfsf1* locus by QTL and genetic analyses. Marker positions refer to the UniSTS annotation for GRCm38. Known protein coding genes within the mapped area are listed at the bottom

Genetic mapping of the *Mfsf1* locus was performed using conventional microsatellite markers and recombinant males to narrow the mapping region obtained by QTL analysis. QTL results indicated that homozygosity of the *Mfsf1*^*B10.M*^ allele was required to express the subfertility phenotype, although the fertility values of the *Mfsf1*^*B10.M*/*B10.M*^ homozygotes varied widely (Fig. 3b). Three informative recombinant males were obtained. The *Mfsf1* locus was mapped between the *D4Mit251* and *D4Mit170* markers by this analysis (Fig. 4a). Together with QTL mapping results, these results indicate that the *Mfsf1* locus is restricted to a 1.53 Mbp region containing 22 protein-coding genes (Fig. 4b). Known information of candidate genes within the mapped interval is listed in Supplemental Table S1.

### Epistatic interactions with the *Mfsf1* locus

**Fig. 5:**
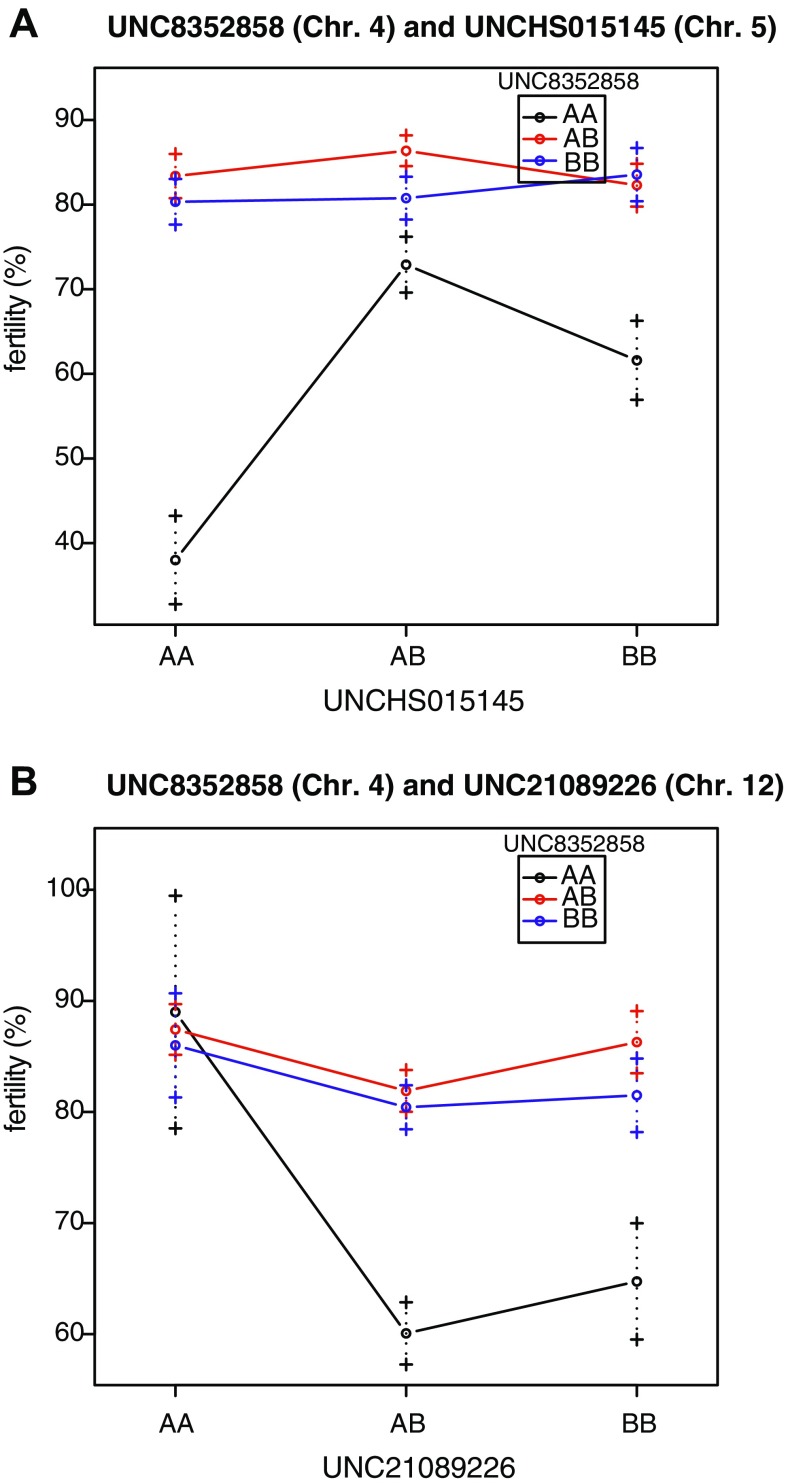
Epistatic effects of factors genetically interacting with the *Mfsf1* locus on fertility. The *UNC8352858* marker represents the nearest marker to the *Mfsf1* locus on chromosome 4. **a** Interaction plot for *UNC8352858* and *UNCHS015145* on chromosome 5. Homozygosity of the *UNCHS025145*^*B10.M*^ allele suppresses fertility. **b** Interaction plot for *UNC8352858* and *UNC21089226* on chromosome 12. Homozygosity of the *UNC21089226*^*B10.M* allele^ enhances fertility. *AA* indicates homozygosity for the B10.M allele, *AB* indicates heterozygosity, and *BB* indicates homozygosity for the C3H allele

The wide distribution of fertility in *Mfsf1*^*B10.M*/*B10.M*^ homozygotes could not be explained by *Mfsf1* genotype alone. No significant QTL peaks showing additive effects were found. Therefore, we next searched for other factors that could interact with the *Mfsf1* locus using the *scantwo* function of the R/qtl package. One significant interacting QTL peak was found on chromosome 5 at 40.13 cM; this locus had a suppressive effect on fertility (Fig. 5a). In addition, one QTL peak with an enhancing effect on fertility was found on chromosome 12 at 20.89 cM. The LOD score for this single QTL peak (2.72) was slightly below the suggestive level (Fig. 5b). No significant interaction of this factor with the *Mfsf1* locus was found.

## Discussion

This study revealed that male subfertility in the B10.M mouse strain is heritable. The *Mfsf1* locus responsible for subfertility is mapped to chromosome 4. It is different from the major locus (*Shm1*) responsible for teratospermia on chromosome 1 (Hirawatari et al. 2015b). We also found that the subfertility phenotype was not associated with the previously reported sperm shape abnormality phenotype in B10.M strain. Similarly, analysis of the TEN1 mouse strain also reported that teratospermia was not associated with male subfertility (Hirawatari et al. 2015a). Male subfertility is often attributed to teratospermia. The results of this study clearly show that sperm shape abnormality phenotype and male subfertility phenotype are not always linked.

Although male subfertility and teratospermia are not associated in B10.M mice, this does not necessarily imply that there is no association between the genes responsible for these two phenotypes. With respect to the teratospermia phenotype in B10.M mice, the two loci responsible, *Shm1* on chromosome 1 and *Shm2* on chromosome 4, have been shown to interact (Gotoh et al. 2012). Homozygosity of the *Shm1*^*B10.M*^ allele is required for expression of the sperm shape phenotype, and the *Shm2*^*B10.M*^ allele enhances the frequency of morphologically abnormal sperm in a recessive manner. The *Shm2*^*B10.M*^ allele alone does not cause teratospermia. The *Shm2* locus has been mapped to chromosome 4 between the *D4Mit148* (69.48 cM, 137148689 bp) and *D4Mit170* (70.47 cM, 138615338 bp) markers. Thus, the two mapped regions, the *Shm2* locus in the teratospermia study and the *Mfsf1* locus in this study, are nearly identical, and the genes encoded by the *Shm2* locus and the *Mfsf1* locus could possibly be identical. Only 22 protein-coding genes are located within the restricted mapped area (Supplemental Table S1).

Because the B10.M strain is an *h2*-congenic strain possessing the *h2*-complex on chromosome 17 in a genetic background derived 75% from C57BL/6 (B6) and 25% from B10 strains (Gotoh 2010), genes on chromosome 4 have originated from either B6 or B10. By searching SNP database, several non-synonymous SNP variants between C57BL/6J and C3H/HeJ strains are found within the coding sequences of the candidate genes (Supplemental Table S2). SNP information of B10 strain for these genes is not available. Because neither B10 males nor C3H males show subfertile phenotype (Hirawatari et al. 2015b), the listed SNP variation is unlikely to cause subfertility of B10.M. A novel mutation appears to have emerged during or after establishment of the B10.M inbred strain. With recent advances in technology, whole genome DNA sequencing is now available for mouse, and genome editing technology has become convenient. These techniques will be required to find genetic variations within the mapped region and then show that the subfertility phenotype can be induced by the identified mutation.

Epistasis, or interaction between genes, was observed in this study. Homozygosity of the *Mfsf1*^*B10.M*^ allele appears to be required to express male subfertility in the B10.M strain, as the other two genotypes, heterozygotes and wild-type homozygotes, exhibit normal fertility. However, less than half of the *Mfsf1*^*B10.M*/*B10.M*^ homozygous males displayed the subfertility phenotype (Fig. 3b). The QTL analysis in this study indicated two bidirectional factors present on chromosomes 5 and 12 that interacted with the *Mfsf1* locus. The locus on chromosome 12 was the only factor found to enhance fertility. Likely due to the limited sample size in this study, the interacting effect of the QTL on chromosome 12 was not statistically significant.

Results from this study demonstrate that genetic approaches can be a powerful tool to analyze mechanisms underlying the complexity of the reproductive system. These approaches are useful as a large number of the 3000 genes expressed in male germ cells may contribute to male infertility (Schultz et al. 2003). In fact, gene knockout studies in mice have already identified 666 genes associated with male infertility (Nagirnaja et al. 2018). In addition, genetic interactions associated with reproductive phenotypes are common. Over the course of evolutionary history, each mammalian species or subspecies has acquired complex mechanisms to regulate normal reproduction. Haldane’s rule in mammals (Coyne 1985) and the hybrid sterility observed between two *Mus musculus* (house mouse) subspecies (Storchova et al. 2004) are two examples of these types of regulatory mechanisms. Another is typified by the frequent appearance of infertile animals among the hybrid descendants of two reproductively normal, inbred mouse strains (Nishimura et al. 1995; Shorter et al. 2017). However, reproductive regulatory mechanisms are not only defined by the extreme condition of infertility, as a wide variety of male reproductive phenotypes has been observed among F2 hybrid males produced by reproductively normal B6 and C3H strains in the absence of a novel mutation (Gotoh and Aoyama 2012). In the genetic study of teratospermia in the TEN1 mouse strain, at least three interacting loci, *Shm3* on chromosome 1, *Shm4* on X chromosome, and *Shm5* on chromosome 6, have been identified (Hirawatari et al. 2015b). In cases in which a single genetic variation causes infertility, genomic methods can be powerful tools for finding the causative gene. However, male reproductive system is supposed to be composed of multiple interactions of genes.

## Electronic supplementary material

Below is the link to the electronic supplementary material.

Supplementary material 1 (DOCX 21 KB)

Supplementary material 2 (DOCX 16 KB)
