# Supplementary material for: Genetic mapping of a male factor subfertility locus on mouse chromosome 4
Source: Mamm Genome. 2018 Aug 31;29(9):663–9. doi: 10.1007/s00335-018-9773-4 (PMC6182756; doi:10.1007/s00335-018-9773-4)
Supplement: Supplementary file 1 — Supplementary material 1 (DOCX 21 KB) [file 335_2018_9773_MOESM1_ESM.docx]

Supplemental Table S1

Candidate genes for *Mfsf1* locus within the mapped interval on Chr. 4

| Start | End | cM |  | MGI ID | Feature Type | Symbol | ^1^Reproductive phenotype | ^2^Testis expression |
| --- | --- | --- | --- | --- | --- | --- | --- | --- |
| 137076595 | 137095101 |  |  | MGI:5590807 | lncRNA gene | *Gm31648* |  |  |
| 137116709 | 137117582 | 69.36 |  | MGI:1921513 | lincRNA gene | *1700037C06Rik* |  |  |
| 137117143 | 137118143 | 69.36 |  | MGI:3702655 | lincRNA gene | *Gm13001* |  |  |
| 137139496 | 137142458 |  |  | MGI:5590921 | lncRNA gene | *Gm31762* |  |  |
| 137159082 | 137165066 | 69.52 |  | MGI:3650099 | lincRNA gene | *Gm13003* |  |  |
| 137171297 | 137178084 |  |  | MGI:5826509 | lincRNA gene | *Gm46872* |  |  |
| 137183987 | 137184062 |  |  | MGI:5455176 | miRNA gene | *Gm25399* |  |  |
| 137184065 | 137184140 |  |  | MGI:5453040 | miRNA gene | *Gm23263* |  |  |
| 137184081 | 137252761 |  |  | MGI:5590979 | lncRNA gene | *Gm31820* |  |  |
| 137277489 | 137299726 | 69.8 |  | MGI:98957 | protein coding gene | *Wnt4* | not known | present |
| 137319696 | 137357720 | 69.83 |  | MGI:106211 | protein coding gene | *Cdc42* | not known | present |
| 137325559 | 137325614 |  |  | MGI:5455549 | snRNA gene | *Gm25772* |  |  |
| 137336386 | 137340094 |  |  | MGI:2441797 | unclassified gene | *A430061O12Rik* |  |  |
| 137350570 | 137352305 |  |  | MGI:1919403 | unclassified gene | *2610020P09Rik* |  |  |
| 137387266 | 137388382 |  |  | MGI:1917225 | lincRNA gene | *2810405F17Rik* |  |  |
| 137401554 | 137409791 | 69.88 |  | MGI:3651647 | protein coding gene | *Cela3a* | not known | present |
| 137420999 | 137430540 | 69.9 |  | MGI:1915118 | protein coding gene | *Cela3b* | not known | present |
| 137436579 | 137438395 | 69.91 |  | MGI:3651180 | antisense lncRNA gene | *Gm13010* |  |  |
| 137453284 | 137455461 | 69.92 |  | MGI:1916630 | protein coding gene | *1700013G24Rik* | not known | present |
| 137468769 | 137570630 | 69.93 |  | MGI:96257 | protein coding gene | *Hspg2* | not known | present |
| 137538286 | 137538370 |  |  | MGI:5530988 | miRNA gene | *Mir7018* |  |  |
| 137572083 | 137574569 | 69.99 |  | MGI:3588210 | protein coding gene | *Ldlrad2* | not known | present |
| 137593755 | 137658537 | 70.0 |  | MGI:2158502 | protein coding gene | *Usp48* | not known | present |
| 137664726 | 137729861 | 70.02 |  | MGI:109338 | protein coding gene | *Rap1gap* | not known | present |
| 137696979 | 137706606 | 70.02 |  | MGI:1917058 | antisense lncRNA gene | *Rap1gapos* |  |  |
| 137741733 | 137796384 | 70.02 |  | MGI:87983 | protein coding gene | *Alpl* | not known | present |
| 137806270 | 137811976 |  |  | MGI:5591238 | lncRNA gene | *Gm32079* |  |  |
| 137862237 | 137965229 | 70.02 |  | MGI:1101357 | protein coding gene | *Ece1* | not known | present |
| 137882370 | 137883937 | 70.02 |  | MGI:3652270 | antisense lncRNA gene | *Gm13012* |  |  |
| 137991841 | 137993607 |  |  | MGI:3035105 | unclassified gene | *BE691133* |  |  |
| 137993022 | 138208508 | 70.02 |  | MGI:1923935 | protein coding gene | *Eif4g3* | not known | present |
| 137994151 | 138027903 |  |  | MGI:5591553 | lncRNA gene | *Gm32394* |  |  |
| 138014003 | 138018382 |  |  | MGI:3611235 | unclassified non-coding RNA gene | *8030494B02Rik* |  |  |
| 138137595 | 138138518 | 70.02 |  | MGI:1920813 | antisense lncRNA gene | *1700095J12Rik* |  |  |
| 138163209 | 138163292 |  |  | MGI:5531049 | miRNA gene | *Mir6399* |  |  |
| 138215714 | 138216189 | 70.03 |  | MGI:1916844 | lincRNA gene | *2310026L22Rik* |  |  |
| 138216296 | 138244683 | 70.03 |  | MGI:109369 | protein coding gene | *Hp1bp3* | not known | present |
| 138250403 | 138261332 | 70.07 |  | MGI:2446215 | protein coding gene | *Sh2d5* | not known | present |
| 138250435 | 138301967 | 70.07 |  | MGI:1098229 | protein coding gene | *Kif17* | not known | present |
| 138304730 | 138312628 | 70.13 |  | MGI:1194508 | protein coding gene | *Ddost* | not known | present |
| 138313409 | 138326307 | 70.14 |  | MGI:1916193 | protein coding gene | *Pink1* | not known | present |
| 138316132 | 138316200 |  |  | MGI:5531329 | miRNA gene | *Mir7019* |  |  |
| 138325980 | 138326089 |  |  | MGI:5531028 | miRNA gene | *Gm27646* |  |  |
| 138338424 | 138367992 | 70.17 |  | MGI:1919519 | protein coding gene | *Cda* | not known | present |
| 138394092 | 138396528 | 70.23 |  | MGI:3651622 | protein coding gene | *Fam43b* | not known | below cutoff |
| 138395198 | 138397714 | 70.23 |  | MGI:1931024 | antisense lncRNA gene | *AB041806* |  |  |
| 138434671 | 138442265 | 70.28 |  | MGI:1915600 | protein coding gene | *Mul1* | not known | present |
| 138454314 | 138460123 | 70.3 |  | MGI:1913509 | protein coding gene | *Camk2n1* | not known | present |
| 138497634 | 138528513 |  |  | MGI:5591708 | lncRNA gene | *Gm32549* |  |  |
| 138565360 | 138635884 | 70.43 |  | MGI:1922968 | protein coding gene | *Vwa5b1* | not known | present |
| 138587624 | 138587729 |  |  | MGI:5452738 | snRNA gene | *Gm22961* |  |  |

Information of candidate genes including protein coding gene, unclassified gene, and non-coding RNA gene is referred to the MGI website (<http://www.informatics.jax.org/marker>).

^1^Reproductive phenotype was searched for in the database of International Mouse Phenotyping Consortium website (http://www.mousephenotype.org) using “reproductive system phenotype” including phenotypes of abnormal male reproductive system physiology and abnormal reproductive system morphology.

^2^Testis expression was searched for in the database of the Expression Atlas of the European Bioinformatics Institute website (<https://www.ebi.ac.uk/gxa/home>).
