# Supplementary material for: Genetic mapping of a male factor subfertility locus on mouse chromosome 4
Source: Mamm Genome. 2018 Aug 31;29(9):663–9. doi: 10.1007/s00335-018-9773-4 (PMC6182756; doi:10.1007/s00335-018-9773-4)
Supplement: Supplementary file 2 — Supplementary material 2 (DOCX 16 KB) [file 335_2018_9773_MOESM2_ESM.docx]

Supplemental Table S2

Coding-NonSynonymous SNPs in the 22 protein coding candidate genes

| Symbol | Start | End | SNP ID (db SNP Build 142) | Map position (GRCm38) | C57BL/6J | C3H/HeJ |
| --- | --- | --- | --- | --- | --- | --- |
| *Wnt4* | 137277489 | 137299726 | not found |  |  |  |
| *Cdc42* | 137319696 | 137357720 | not found |  |  |  |
| *Cela3a* | 137401554 | 137409791 | rs16811835 | 137405900 | C | T |
| *Cela3b* | 137420999 | 137430540 | not found |  |  |  |
| *1700013G24Rik* | 137453284 | 137455461 | not found |  |  |  |
| *Hspg2* | 137468769 | 137570630 | rs27576908 | 137518953 | C | T |
|  |  |  | rs27576791 | 137544464 | A | G |
|  |  |  | rs27561735 | 137553740 | C | T |
|  |  |  | rs27561734 | 137553751 | G | C |
| *Ldlrad2* | 137572083 | 137574569 | not found |  |  |  |
| *Usp48* | 137593755 | 137658537 | not found |  |  |  |
| *Rap1gap* | 137664726 | 137729861 | not found |  |  |  |
| *Alpl* | 137741733 | 137796384 | not found |  |  |  |
| *Ece1* | 137862237 | 137965229 | rs27545386 | 137921157 | A | G |
| *Eif4g3* | 137993022 | 138208508 | not found |  |  |  |
| *Hp1bp3* | 138216296 | 138244683 | not found |  |  |  |
| *Sh2d5* | 138250403 | 138261332 | not found |  |  |  |
| *Kif17* | 138250435 | 138301967 | rs27545790 | 138288063 | A | G |
| *Ddost* | 138304730 | 138312628 | not found |  |  |  |
| *Pink1* | 138313409 | 138326307 | not found |  |  |  |
| *Cda* | 138338424 | 138367992 | not found |  |  |  |
| *Fam43b* | 138394092 | 138396528 | not found |  |  |  |
| *Mul1* | 138434671 | 138442265 | rs27576633 | 138439345 | A | G |
| *Camk2n1* | 138454314 | 138460123 | not found |  |  |  |
| *Vwa5b1* | 138565360 | 138635884 | not found |  |  |  |

No coding-nonynonymous SNP is reported for the C57BL/10J, C57BL/J, nor C3H/He strains..
